# Mitochondrial dysfunction at the intersection of alcohol use disorder and chronic pain

**DOI:** 10.1152/function.091.2025

**Published:** 2026-01-30

**Authors:** Xavier R. Chapa-Dubocq, Scott Edwards

**Affiliations:** Department of Physiology, Alcohol and Drug Abuse Center of Excellence, Comprehensive Alcohol—HIV/AIDS Research Center, Louisiana State University Health Sciences Center, New Orleans, Louisiana, United States

**Keywords:** alcohol, bioenergetics, glucocorticoid, mitochondria, pain

## Abstract

Alcohol use for pain relief dates back centuries. This profound analgesic efficacy also represents a strong motivational force that drives excessive drinking, fostering the development and severity of alcohol use disorder (AUD) in vulnerable individuals. Paradoxically, excessive alcohol drinking contributes to a multifactorial neuropathy, increasing nociceptive sensitivity (termed hyperalgesia) and pain-related negative affect, which may promote further alcohol use to manage either preexisting or newly emerging pain symptoms via stress-related neural damage and potentiation of negative reinforcement behavioral systems. These close relationships reflect the urgent need for better research conceptualizations and translational successes for the treatment of both chronic pain and addiction-related disorders. Mitochondrial health is particularly important across critical networks of neurons and nociceptive fibers, where continuous bioenergetic supply is required for axonal transport, repair, and synaptic transmission. Specific bioenergetic mechanisms underlying peripheral nerve damage and subsequent central nervous system adaptations in functional association with pain and excessive alcohol drinking are starting to be discovered. This focused review proposes that mitochondrial damage may unify several convergent pathophysiological mechanisms known to manifest in the context of both chronic pain and AUD and to be particularly relevant for vulnerable patient populations such as persons living with human immunodeficiency virus (HIV). Future research directions aimed at developing and testing novel therapeutic avenues to support mitochondrial health may provide safer and more effective medications for the management of both chronic pain states and AUD.

## Graphical abstract

**Figure:**
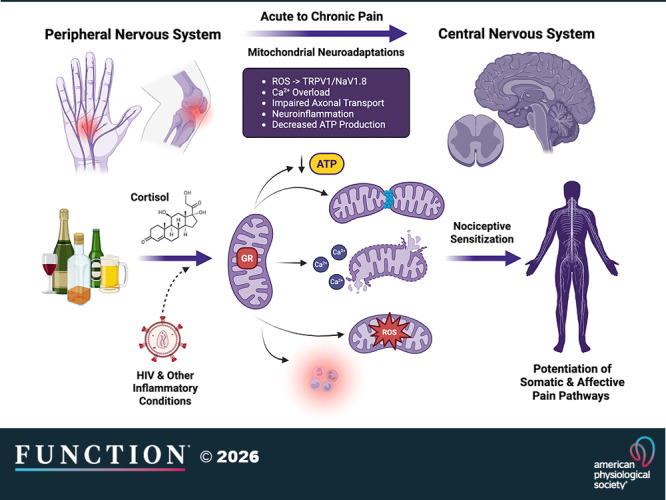

## THE PATHOPHYSIOLOGICAL AND SOCIETAL IMPACTS OF ALCOHOL USE

Alcohol is one of the most widely available and frequently used recreational substances. Although most individuals are able to control their drinking, many exhibit an escalation to binge drinking, a pattern of alcohol consumption that elevates blood alcohol levels (BALs) at or above 0.08% (80 mg/dL), generally resulting from consumption of 4–5 standard alcohol drinks over 2 h (1). It is critical that individuals limit their drinking below these levels, and although there are likely no safe levels of alcohol use, the biomedical consequences of binge drinking in particular impair multiple systemic aspects of cellular physiology and metabolism and impact virtually every major tissue and organ system in the body (2). From a mental health perspective, excessive alcohol use damages both the peripheral and central nervous systems over time, manifesting in a constellation of neurological and psychiatric conditions, including alcohol use disorder (AUD). Beyond damaging physiological systems, AUD produces additional psychosocial costs to individuals and society and is formally defined within the Diagnostic and Statistical Manual of Mental Disorders, Fifth Edition (DSM-5). AUD is also a progressive, longitudinal disorder characterized by escalation of alcohol consumption, loss of control over drinking, continued use despite several personal and health consequences, high propensity to relapse during attempted abstinence, and the emergence of negative affective symptoms during withdrawal or attempted abstinence (3). Negative reinforcement theories of AUD posit that individuals use alcohol in attempts to self-medicate such negative motivational states, and that strategies aimed at reducing alcohol use will cascade to benefit the overall health of individuals and society.

## CRITICAL LINKS BETWEEN ALCOHOL USE AND CHRONIC PAIN

As one major construct of negative affect that influences alcohol drinking, pain impacts 27% of the global adult population (4). Unrelieved pain represents a harmful subjective experience that powerfully influences brain reward and reinforcement mechanisms, possibly facilitating the transition to AUD and other substance use disorders in vulnerable individuals (5). A meta-analysis of controlled empirical laboratory studies discovered that alcohol serves to relieve pain, although effective analgesia is observed near and above the established binge drinking limits that foster risk of AUD (6). Moreover, and in contrast to the acute analgesic benefits, chronic alcohol use often contributes to increased pain sensitivity as part of a multifactorial neuropathy and a more widespread alcohol withdrawal syndrome (3, 7, 8). Indeed, problem drinkers of both sexes report more severe pain symptoms compared with nondrinkers and also a higher incidence of using alcohol to manage their pain (9). Pain is also a strong predictor of relapse to drinking both during and after AUD treatment, further signifying pain as a significant driver of AUD severity (10). Thus, neurophysiological interactions of pain and excessive alcohol drinking represent a critical area of research and public health interest to develop novel pain interventions that serve as safer alternatives to alcohol (11).

## MITOCHONDRIAL DYSFUNCTION IN THE CONTEXT OF CHRONIC PAIN AND ALCOHOL DRINKING

Unfortunately, very few treatments for multiple chronic pain conditions are available. Despite significant investment and research worldwide, the basic pathophysiology of chronic pain is still incompletely understood, and the translational impact of preclinical discoveries in pain research has been discouraging (12, 13). Innovative research conceptualizations that incorporate a deeper biological understanding of nociception and pain pathophysiology are desperately needed. Trends in our greater understanding of cellular bioenergetics and the critical nature of mitochondrial health to neurological and psychiatric wellness have recently emerged (14, 15).

Mitochondria serve as more than just cellular powerhouses; they are central regulators of bioenergetics, redox signaling, calcium homeostasis, and apoptotic cascades, all of which are critical for maintaining neuronal excitability and synaptic plasticity (16). Mounting evidence has positioned mitochondrial dysfunction as a key driver in the development and persistence of chronic pain, including neuropathic and inflammatory states (17–19). Excessive production of mitochondrial reactive oxygen species (ROS) not only damages macromolecules but also sensitizes peripheral nociceptors, thereby amplifying pain signaling and fueling a pathological feedback loop between oxidative stress and inflammation (20, 21). Although ROS are important for mitochondrial homeostasis and signal transduction, overproduction (sometimes resulting from the loss of endogenous antioxidant buffering capacity) can produce mitochondrial damage and irreversible cellular dysfunction. ROS include highly reactive species (e.g., hydroxyl radical) and moderately reactive species (e.g., superoxide) that are the by-products of oxidative phosphorylation. Increased spinal production of the free radical nitric oxide (considered a reactive nitrogen species) is also elevated in animal models of neuropathic pain (22). Furthermore, nitric oxide and superoxide can combine to form peroxynitrite, another oxidant and nitrating agent implicated in persistent pain states (23). The relative contributions of various ROS to oxidative stress in chronic pain states have been difficult to measure, and cumulative ROS generation (both baseline and stimulated) is commonly assayed via fluorescent makers such as MitoTracker Red (24). Activation of superoxide dismutase (SOD) by systemic or intrathecal administration of SOD small molecule mimetics such as M40403 (imisopasem manganese) has long been known to provide pain relief independent of the opioid system (25), and early studies discovered a critical role for spinal dorsal horn mitochondrial antioxidants in the regulation of persistent pain states (26). Systemic administration of ROS scavengers in the context of hyperalgesia has also been shown to reduce hallmarks of central sensitization in dorsal horn neurons (27). Medication strategies aimed at reducing SOD nitration or superoxide generation have therefore been proposed as novel analgesics (28). Alcohol consumption adds another layer of complexity, as it independently perturbs mitochondrial health and has been shown to worsen pain syndromes (29–31) and strengthen ties between pain and negative affect (32). Given the intersection between alcohol use, mitochondrial health, and chronic pain, this section will cover the current understanding of mitochondrial bioenergetics and dysfunction in pain states, with particular attention to alcohol-exacerbated modulation across both peripheral and central nervous systems.

Mitochondrial dysfunction plays a pivotal role in central sensitization, a core feature of chronic pain, characterized by heightened neuronal excitability and maladaptive synaptic plasticity across key nervous system pain circuits (33–36). In the spinal dorsal horn, deficits in mitochondrial calcium buffering and ATP production facilitate long-term potentiation and persistent nociceptive signaling (37, 38). Beyond the spinal cord, chronic alcohol exposure produces profound mitochondrial architectural and bioenergetic disturbances within higher-order brain regions such as the amygdala and prefrontal cortex, areas critical for integrating nociceptive input with emotional and cognitive processing (39, 40). Mitochondrial impairment in these regions hinders the homeostatic balance of inhibitory and excitatory neurotransmission, thereby exacerbating pain perception and emotional dysregulation. Similarly, mitochondrial dysfunction in the thalamus may intensify nociceptive relay and central amplification (41). Although these circuit-level alterations highlight the broad impact of mitochondrial dysfunction on pain processing, alcohol further exacerbates cellular vulnerability by directly targeting mitochondrial integrity.

Peripheral sensory neurons are highly vulnerable to mitochondrial dysfunction due to their large metabolic demands and dependence on sustained axonal transport for long-range signaling. Mitochondria provide the ATP necessary for excitability, vesicular trafficking, and axonal integrity, making their health essential for nociceptive processing. When mitochondrial function deteriorates, as observed across multiple animal models of peripheral neuropathy and inflammatory arthritis pain, energy failure and oxidative stress drive hyperalgesia and allodynia (19, 42–45). A key mechanism is excessive ROS production, which sensitizes TRPV1 and Nav1.8 channels, increases calcium and sodium influx, and heightens neuronal excitability (46–48). Calcium dysregulation serves as a crucial pathological link between mitochondrial dysfunction and heightened pain sensitivity. Under physiological conditions, mitochondria act as buffers for intracellular calcium, preserving neuronal excitability within functional limits (49). Chronic alcohol exposure impairs this buffering capacity, reducing mitochondrial calcium uptake and leading to elevated cytosolic calcium levels (50, 51). This calcium overload activates degradative enzymes that exacerbate neuronal injury and sensitize both peripheral and central pain pathways (52, 53). Mitochondrial calcium dysregulation could be especially relevant in the context of alcohol dependence, where excessive mitochondrial calcium uptake can contribute to nociceptive hypersensitivity.

The mitochondrial calcium uniporter (MCU) governs calcium influx into mitochondria and plays a critical role in regulating oxidative phosphorylation (54–56). However, under pathological conditions, calcium overload prompts the opening of the mitochondrial permeability transition pore (mPTP), resulting in mitochondrial depolarization, ATP depletion, and eventual cell death via apoptosis or necrosis (57, 58). Once activated, the mPTP becomes permeable to solutes up to 1.5 kDa, disturbing mitochondrial homeostasis through osmotic swelling and membrane rupture. Cyclophilin D (CypD) is a known regulator of mPTP opening, although its molecular configuration remains incompletely defined (59). Dysregulated mPTP activity is manifested during alcohol withdrawal, likely contributing to increased neuronal excitability, enhanced neuroinflammatory signaling, and pain-related symptoms (60, 61). These effects create a feedback loop that further inhibits mitochondrial calcium homeostasis and augments nerve damage (58, 62, 63) that may facilitate neuropathic pain symptoms. One critical mechanism involves dysregulation of the mPTP, which serves as a pivotal gateway between adaptive stress responses and irreversible cell death pathways. Chronic alcohol consumption increases mPTP activity, triggering both apoptotic and necrotic pathways and elevating proinflammatory signaling cascades (64, 65). Importantly, pharmacological inhibition of mPTP has been shown to attenuate alcohol-induced mitochondrial damage, underscoring its potential as a therapeutic target for alcohol-exacerbated hyperalgesia (66).

Beyond its role in initiating cell death, persistent mPTP opening also compromises mitochondrial bioenergetics by collapsing the proton gradient, thereby limiting ATP synthesis. ATP depletion is especially detrimental in neurons and nociceptive fibers, where continuous energy supply is required for axonal transport, repair, and synaptic transmission (67–69). Prolonged energy failure further triggers apoptotic cascades through cytochrome c release and caspase activation, ultimately leading to sensory neuron degeneration (70, 71). Alcohol further exacerbates this pathology by inhibiting electron transport chain activity (31, 72), reducing β-oxidation, and impairing complexes I and V in nerve tissue, thereby compounding ATP deficits and oxidative stress (73). These alterations undermine neuronal resilience. Importantly, nonneuronal cells contribute as well: mitochondrial dysfunction in Schwann cells impairs myelin maintenance and trophic support, accelerating axonal degeneration (74). Moreover, alcohol-related metabolic deficiencies such as thiamine depletion increase peripheral nerve vulnerability (75). Collectively, these factors converge to heighten metabolic stress and promote progressive neurodegeneration. Finally, oxidative stress itself promotes mitochondrial fragmentation (76), further destabilizing peripheral and central nervous systems after regular exposure to alcohol.

Chronic alcohol exposure introduces further direct insults to mitochondrial health. Alcohol has been shown to induce mitochondrial swelling, fragmentation, and enzymatic inhibition, leading to reduced energy production and increased oxidative burden (77). The fragmentation of mitochondria facilitates healing by enabling their transport along axons for fusion and material exchange, whereas damaged organelles are returned to the cell body via retrograde transport in autophagosomes for degradation. However, when proper axonal transport is inhibited, whether by impaired anterograde delivery that limits mitochondrial availability at synapses or by reduced retrograde clearance of damaged organelles, it can drive mitochondrial dysfunction and contribute to altered pain sensitization. Supporting this concept, alcohol exposure has been shown to impair vesicular transport in neurons; specifically, retrograde tracing of dye injected into the sciatic nerve demonstrated reduced transport efficiency in ethanol-fed rats (78–80). Consistently, electron microscopy following alcohol exposure revealed abnormal accumulations of organelles, including mitochondria, clear vesicles of varying sizes, and dense vesicles, further indicating impaired axonal trafficking (81). Recently, in vivo, two-photon time-lapse imaging revealed that acute ethanol intoxication markedly increased mitochondrial mobility, indicating a newly disordered trafficking and positioning that is normally essential for sustaining synaptic stability and energy supply (82). Such alterations in mitochondrial dynamics may compromise neuronal resilience and contribute to maladaptive plasticity underlying altered pain sensitization. Mitochondrial fission, driven by the GTPase Drp1, is essential for organelle quality control and distribution but becomes pathological when excessive. Inhibition of Drp1, either by antisense knockdown or the selective fission inhibitor mdivi-1, attenuates ddC- and oxaliplatin-induced mechanical hyperalgesia in a rodent model, underscoring its role in neuropathic pain (83). Since alcohol also perturbs mitochondrial dynamics and promotes oxidative stress, Drp1-mediated fission may represent a key mechanism in alcohol-exacerbated pain states. Beyond their roles in ROS generation and fission, mitochondria also function as signaling hubs that regulate innate immune responses, linking metabolic stress to neuroinflammation (84).

Neuroinflammation is increasingly recognized as a critical mediator linking mitochondrial dysfunction to pathological pain perception. Alcohol consumption activates glial cells, particularly microglia and astrocytes, which release proinflammatory cytokines that impair mitochondrial bioenergetics and increase ROS production (30, 85). This proinflammatory environment impairs proper synaptic function and facilitates maladaptive plasticity, contributing to the persistence of chronic pain behaviors (86). Oxidative stress lies at the core of this cycle. Alcohol metabolism and alcohol-induced mitochondrial dysfunction lead to antioxidant depletion and excessive formation of ROS (87), including the standard ROS and the unique, ethanol-derived 1-hydroxyethyl radical (88). Alcohol-induced ROS can damage cellular lipids, proteins, and DNA while also activating membrane channels and intracellular signaling cascades that may impact nociceptive thresholds (89–91). The mitochondrial antiviral signaling (MAVS) proteins, located in the outer membrane, detect cytosolic viral double-stranded RNA and activate NF-κB and IRF3, driving proinflammatory cytokine and type I interferon production (92, 93). Activation of NF-κB signaling extends beyond innate immune responses, as this pathway is markedly upregulated in reactive astrocytes and contributes to the maintenance of neuroinflammation and neuropathic pain (94). Therefore, modulation of mitochondrial control over NF-κB activation represents a potential therapeutic avenue to mitigate inflammation-driven pain states (95, 96). In summary, heightened inflammatory signaling can sensitize nociceptive pathways, lower pain thresholds, and contribute to chronic pain states. Additional preclinical and translational research into alcohol’s effects on mitochondrial-immune interactions is warranted to discover safe and effective interventions targeting these mechanisms. A key intersectional neuroendocrine network dysregulated in the context of these conditions is the glucocorticoid system, and these connections are expanded upon in the next section.

## STRESS-RELATED GLUCOCORTICOID SYSTEM DYSFUNCTION AND MITOCHONDRIAL DAMAGE

Decades of translational research has described pathological glucocorticoid activity in the context of AUD, manifesting both at the level of the hypothalamic-pituitary-adrenal (HPA) axis (97, 98) and within key brain areas such as the central amygdala (99, 100), described further in the next section. The excessive activation of brain glucocorticoid receptors (GRs) is posited to facilitate cognitive/executive dysfunction, negative emotional states, and escalated alcohol consumption, all of which are common features of AUD (99, 101). Representing the major neuroendocrine circuit regulating production of the major stress hormone cortisol, dysregulation of the HPA axis is another frequent and convergent finding in individuals suffering signaling across various stages of AUD, characterized by increased basal cortisol levels and altered HPA axis activation in response to stress exposure (102, 103).

Importantly, but perhaps less well described, heightened GR activity may also play a role in alcohol-related neuropathy and AUD-associated hyperalgesia. Support for this hypothesis is based on consistent evidence that mifepristone, a GR antagonist, both prevents and reverses the hyperalgesia associated with alcohol withdrawal (104). At the same time, mifepristone administration in rats and humans blocks the escalation of alcohol drinking and craving symptoms (105, 106). As glucocorticoids signal across various crucial genomic and nongenomic mechanistic pathways, it is perhaps not surprising that chronic stress produces behavioral adaptations in direct relation to mitochondrial function along the HPA axis in preclinical animal models (107). Indeed, several links exist between stress-related glucocorticoid activity and cellular bioenergetics in the nervous system, leading to the description of mitochondria as endocrine organelles and allostatic load stress as an energy-driven process (108, 109). Mitochondria house the rate-limiting enzymes for glucocorticoid synthesis (110). Glucocorticoids also dynamically regulate neuronal mitochondrial functions in a classic inverted “U”-shape (111), with low doses facilitating synaptic strength and being neuroprotective and chronic, high-dose exposure producing neurodegeneration.

## NERVOUS SYSTEM VULNERABILITY TO CHRONIC STRESS- AND PAIN-RELATED MITOCHONDRIAL DYSFUNCTION

As discussed earlier, although excessive alcohol exposure damages elements of the peripheral nervous system (PNS) to exacerbate neuropathic pain symptoms, the resulting chronic and persistent hyperalgesia is hypothesized to drive motivational factors to consume alcohol based on its analgesic properties. This association essentially describes the behavioral process of negative reinforcement, believed to be critical for the transition from recreational drinking to AUD. Both chronic stress and excessive alcohol use also directly damage highly specialized central nervous system (CNS) pain- and cognition-related circuitry and potentiates ascending nociceptive circuitry to produce enduring cognitive deficits and affective pain symptoms that manifest in the context of more severe forms of AUD. Although drug-induced neuroadaptations typically associate with initial plasticity changes in the mesolimbic dopamine system, these will eventually extend to elements of what is termed the extended amygdala, including the central amygdala (112). Over time, repeated activation of these circuits results in dysregulation of motivated behavior.

Historical investigations into chronic pain pathophysiology have focused their attention on peripheral and spinal aspects of nociception, where numerous plastic changes have been described in relation to a central sensitization conceptualization of the transition from acute to chronic pain states (113). Other investigators have criticized the insufficiency of spinal mechanisms to explain pain and pain relief (114). Important to this critique, nociceptive information ascends to higher brain centers where the subjective experience of pain emerges as a multidimensional construct. In addition to somatosensory elements, both affective/emotional and cognitive/motivational dimensions exist that augment chronic pain-related morbidity (40). Unrelieved pain generates a continuous negative affective state and promotes a reorganization of cognitive/motivational strategies to avoid pain. As a consequence, the relief of pain itself activates brain reinforcement circuitry and is experienced as rewarding (115). Such negative reinforcement processes motivate individuals to seek relief from pain by escalating use of alcohol and other analgesics, culminating in the risk of psychiatric sequelae including alcohol and other related substance use disorders (116, 117).

Preclinical evidence also highlights the importance of central stress hormone signaling in the chronification of pain. Systemic administration of synthetic glucocorticoids has long been used clinically for the management of inflammatory states often associated with pain, as GR transcriptionally regulates the balance of pro- and anti-inflammatory mediators in circulating immune cells that target the site of injury (118). However, long-term exposure to glucocorticoids can lead to antinociceptive tolerance and may begin to promote the engagement of pronociceptive systems in a phenomenon termed stress-induced hyperalgesic priming (119, 120). GR-related neuroplasticity within the CNS (both spinal cord and brain) appears to be key to these processes (121). In addition to GR, closely related signaling pathways such as serum glucocorticoid-regulated kinase 1 (SGK1) have also been implicated in the development of chronic pain (122) and analgesic tolerance (123) through heightened activity in the spinal cord. Research suggests that chronic pain is closely associated with potentiation of central amygdala stress systems (124) and that cortisol functions directly within this limbic brain region to increase visceral and somatic hypersensitivity by increasing expression (124) and activity of the stress neuropeptide hormone corticotropin-releasing factor (CRF) (125). A similar mechanism may underlie more complex neuropathological conditions that engage higher brain functions, such as hyperalgesia and escalated alcohol use produced by past traumatic stress exposure since these conditions are reduced by administration of CRF1 receptor antagonists directly into the central amygdala (126–128).

Based on their central role at the intersection of affective pain and cognition, the elucidation of mitochondrial and bioenergetic neuroadaptations in central brain reward/reinforcement and higher nociceptive areas may provide additional mechanistic insights into more effective treatments for both chronic pain and pain-related negative affect in the context of AUD. A recent study found that knockdown of mitochondrial trafficking in dopaminergic neurons abolished conditioned alcohol preference in *Drosophila melanogaster* (82), and such evidence further suggests that mitochondrial damage not only damages the brain but may represent a key process in reward and reinforcement learning. Central brain changes in mitochondrial plasticity are also evident. For example, mitochondrial ROS play a role in the manifestation of pain-related behaviors within the central amygdala (129). Given the presence of functional glucocorticoid receptors in the mitochondria (111, 130, 131), a remarkable but largely underexplored area of investigation is GR regulation of mitochondrial function in chronic pain states. Preclinical models of social isolation stress link increased mitochondrial GR phosphorylation to proapoptotic signaling in the prefrontal cortex (132). Additional research has indicated that neuroinflammation-induced depression-like behaviors are also related to mitochondrial GR function (133). A key gap in the literature is how to dissociate GR-related mitochondrial dysfunction in chronic pain states versus the negative affective experiences that may naturally emerge in the context of unresolved pain. The impairment of several mitochondrial quality control (MQC) systems by glucocorticoids (134) would likely play a central role in the manifestation of both chronic pain and negative affective consequences. A closer investigation of how each of these specific MQC mechanisms (ranging from disruption of cellular bioenergetics to mitochondrial trafficking and turnover) is dysregulated across both peripheral and central nervous systems is worth future exploration in preclinical pain and AUD models.

## PEOPLE WITH HIV AS AN ILLUSTRATIVE POPULATION

**Figure 1.**
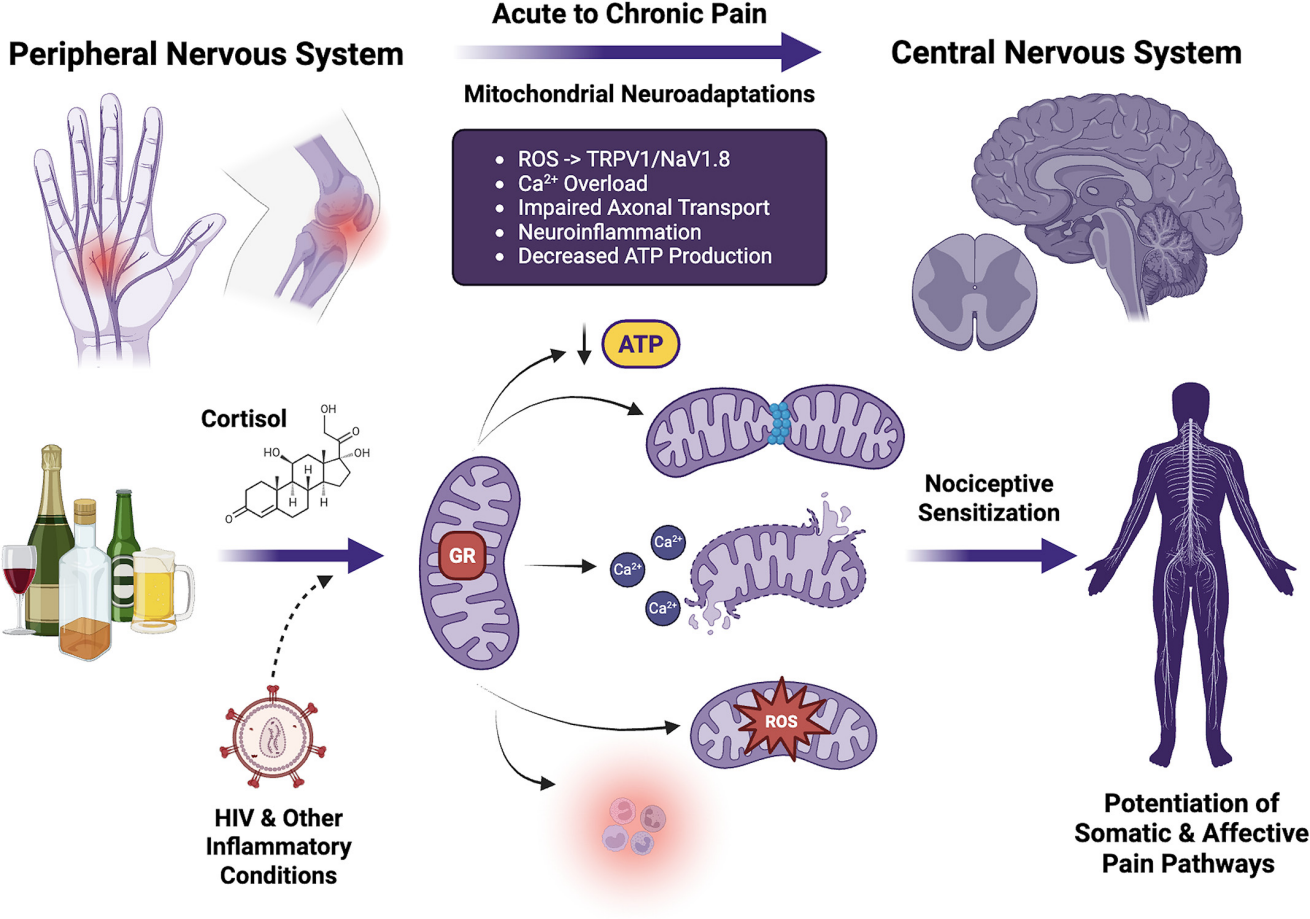
Mitochondrial dysfunction as a convergent mechanism across alcohol-and stress-exacerbated pain states. Alcohol use and inflammatory conditions such as HIV infection converge on mitochondria to impair ATP production, calcium buffering, and redox balance, and the effects may be further exacerbated by stress-associated glucocorticoid receptor (GR) activity. These alterations drive excessive reactive oxygen species (ROS), calcium overload, mPTP opening, and impaired axonal transport, which collectively promote neuroinflammation and neuronal injury. In the peripheral nervous system (PNS), mitochondrial dysfunction sensitizes nociceptive ion channels (e.g., TRPV1, NaV1.8), impairs axonal transport, and reduces energy availability, leading to hyperexcitability and heightened nociceptive transmission into the CNS. Within the CNS, similar mitochondrial deficits contribute to central sensitization, excitatory-inhibitory imbalance, and maladaptive synaptic plasticity in higher brain affective and reward/reinforcement centers such as the central amygdala. Together, these mechanisms highlight mitochondria as a shared pathological nexus through which alcohol and stress exacerbate pain vulnerability. Figure created with a licensed version of BioRender.com.

The already widespread prevalence of chronic pain is expected to increase over the coming decades as our global population ages. Successful aging will rely on maintaining and even accelerating the quality of life of individuals who live longer but suffer from polymorbid conditions. A good example of this transition is the current status of people aging with human immunodeficiency virus (HIV). Although more effective viral suppression through antiretroviral therapy has prevented the progression to acquired immune deficiency syndrome (AIDS), people with HIV (PWH) are hypothesized to still experience the phenomenon of “inflammaging” over time (135), driving new investigations into how inflammation-related comorbidities may hinder successful aging (136). At-risk alcohol use is among the principal maladaptive coping behaviors in PWH, and AUD frequently occurs in this vulnerable population (137). Similar to alcohol, inflammatory conditions such as HIV infection can also produce a characteristic neuropathy (138) that further alters pain sensitivity in the context of alcohol consumption, in part through mitochondrial dysfunction. Pain severity is associated with greater drinking to manage pain and dissatisfaction with pain care (139), a relationship that may be driven by alcohol-induced mitochondrial dysfunction exacerbating both nociceptive signaling and neuroinflammation processes. Chronic pain and hazardous alcohol use jointly heighten mitochondrial vulnerability, as shown by increased circulating mitochondrial DAMPs after painful stress, with HIV status potentially modifying this effect (21). Indeed, a preclinical study revealed how the HIV gp120 coat protein mediated a transition from acute to chronic pain in association with mitochondrial superoxide changes in the spinal cord of rats (140). Furthermore, in people with HIV, high-risk alcohol use intensifies stress-induced mitochondrial damage, underscoring heightened vulnerability of mitochondrial health in this population (29). This vulnerability may extend beyond pain to neuropsychiatric conditions such as depression and anxiety, which are frequently comorbid with chronic pain and AUD (32, 141). Ongoing alcohol use also appears to strengthen relationships between cortisol and negative affect in PWH (142). Alcohol consumption was also shown to increase highly active antiretroviral therapy (HAART)-induced neuropathic pain via mitochondrial mechanisms in a preclinical rodent model (143). Although the antiretroviral therapy (ART) investigated in that study [dideoxycytidine (ddt)] is no longer regularly used in PWH, it is possible that PWH were impacted by past ART exposure, and additional research is needed to investigate the impact of more modern ART regimens (144). Together, these findings indicate that HIV/ART, pain, and alcohol use converge on mitochondrial dysfunction as a shared mechanism of vulnerability (Fig. 1).

## SUMMARY AND FUTURE DIRECTIONS

Strong bidirectional relationships between AUD and chronic pain have been discovered (40, 145–147) whereby pain and pain-related negative affect may represent a significant and cumulative allostatic stress load that greatly impacts the individual and society as a whole. Few effective therapies exist for either AUD or chronic pain. The accretive pathophysiology and shared neurophysiological interactions of these disease states likely complicate their effective treatment. Powerful negative reinforcement processes promote and maintain alcohol drinking to manage pain and the negative emotional states that underlie chronic pain experiences. Future funding and research priorities should aim to bridge gaps in our understanding of how alcohol acts on nociceptive and higher motivational circuitry to drive hyperalgesia symptoms that may exacerbate AUD. Numerous symptoms regularly associate with severe AUD, ranging from poor risk management to the cognitive/affective dimension of pain, are likely driven by neuroadaptations within key anatomical elements that regulate higher executive functions, including key contributions from the central amygdala. Additional research is needed to examine roles for mitochondria in supporting the effective bioenergetics of healthy versus diseased circuit functions within these key systems (148).

An enormous amount of justified scientific and clinical attention has been paid to the challenge of maximizing effective pain relief while reducing addiction liability of illicit and prescription opioid medications. The search for novel, nonopioid analgesics has been steadily progressing, with individuals even becoming open to the potential analgesic utility of additional controlled psychoactive substances like medicinal cannabis (149). It is important to understand the desperate situation that chronic pain patients and their care providers find themselves in and the wide range of pharmacological and behavioral strategies (both beneficial and harmful) that may be implemented in attempts to achieve some relief from continuous pain and the emotional distress that accompanies pain. Consequently, a greater understanding of polysubstance abuse, biobehavioral mechanisms of analgesic cross tolerance, and other aspects of polypharmacy on stress-related pathophysiology and mitochondrial health and dynamics is warranted. For example, individuals with a history of opioid use disorder (OUD) exhibit a significant upregulation of GR pathway genes within the central amygdala compared with healthy controls (150), suggesting that GR dysregulation may extend to other widely misused analgesic substances such as opioids. The opioid morphine also impairs brain mitochondrial function and produces oxidative stress (151–153). A critical question is whether a safe pharmacological strategy could be developed that restores mitochondrial health while offering an analgesic alternative to alcohol and opioids. As one highly promising example, a series of preclinical and clinical studies have demonstrated the widespread benefits of supplementation with mitoquinone (MitoQ) to improve cardiometabolic health (154). Although one animal study also demonstrated a potential benefit of mitoquinone to alleviate neuropathic pain symptoms and mitochondrial damage in a vincristine chemotherapy model (155), additional work is needed to determine whether mitochondria-targeted antioxidants represent a viable therapeutic strategy for other chronic pain conditions.

Finally, the vital contributions of sex as a biological factor in AUD risk, pain sensation, and related negative affect are also beginning to be appreciated (156). Cortisol impacts amygdala functional connectivity to other nociceptive areas such as the cingulate cortex in a sex-specific manner (157), highlighting just one of the several established sex differences in the neurobiology and susceptibility to neurological and psychiatric disease, including potentially important relationships between steroid hormones and mitochondrial bioenergetics (158, 159). Findings from preclinical animal models suggest that mechanistic sex differences may also exist with regard to interventions aimed at supporting mitochondrial health. A recent study found that aged male and female mouse hearts appear phenotypically different with regard to the beneficial effects of glutathione supplementation and corresponding functional (exercise-based) outcomes (160). Brain mitochondrial bioenergetics also appear to be altered by diet in a sex-specific manner (161). These studies caution that our ability to develop effective lifestyle and pharmacological interventions to improve mitochondrial health will likely require additional basic science research to discover mechanistic insights to inform translational efforts. Given the central role of mitochondria in supporting nervous system function, these investments will no doubt pay enormous dividends for the future benefit of vulnerable individuals at risk for AUD and chronic pain.
